# Superior Capsular Release After Failed Combined Superior Labral Repair And Biceps Tenodesis For Slap Tear

**DOI:** 10.2174/1874325001812010295

**Published:** 2018-07-31

**Authors:** Yung Han, Janet Lee, Sung Park, Eugene Suh

**Affiliations:** Los Angeles Shoulder Institute, 505 S Virgil Ave, Ste. 205, Los Angeles, CA 90020, USA

**Keywords:** Slap Tear, Biceps Tenodesis, Superior Capsule Release, Failed Slap Repair, Shoulder Stiffness

## Abstract

**Introduction:**

Optimal treatment of type II superior labrum anterior and posterior (SLAP) tears is controversial. There has been a recent trend towards biceps tenodesis over SLAP repair in older patients. Few surgeons have performed combined biceps tenodesis and SLAP repair with inferior results.

**Case Report:**

This case describes a 46-year-old patient who had persistent pain and stiffness after combined biceps tenodesis and SLAP repair for a type II SLAP tear. His pain and motion improved after arthroscopic superior capsular release.

**Conclusion:**

Failed SLAP repair is often multifactorial and a thorough workup is needed. Combined biceps tenodesis and SLAP repair can cause pain, stiffness, and dysfunction which can be successfully treated with arthroscopic superior capsular release.

## 1 INTRODUCTION

Type II superior labrum anterior and posterior (SLAP) repairs have historically been repaired and have been fraught with mixed results [1-3] and complications [4-7]. There has been a recent trend towards biceps tenodesis for the surgical management of type II tears [8, 9] and treatment of failed SLAP repairs [10-12]. Few surgeons have performed combined biceps tenodesis and SLAP repair to treat biceps pathology and optimize glenohumeral biomechanics [13]. However, a recent study showed that this combined procedure results in significantly worse forward flexion and pain scores compared to isolated SLAP repair or isolated biceps tenodesis [13]. We present a patient who had persistent pain, stiffness, and dysfunction after receiving concomitant biceps tenodesis and superior labral repair. His pain, stiffness, and function improved after arthroscopic superior capsular release (including release of the Superior Glenohumeral Ligament (SGHL), Coracohumeral Ligament (CHL), Middle Glenohumeral Ligament (MGHL), Rotator Interval (RI)) and subacromial decompression.

## 2 CASE PRESENTATION

The patient is a 46-year-old right-hand dominant male who presented for left shoulder pain, stiffness, and mechanical symptoms. He underwent arthroscopic SLAP repair and open subpectoral biceps tenodesis 2 years ago for long-standing left shoulder pain without any history of trauma. He stated that his pain and dysfunction were worse at this time than before the index surgery. Golf was his main recreational activity before the surgery, but now he has problems doing activities of daily living. He has not improved with physical therapy. He has been to two other orthopaedic surgeons and was diagnosed with subacromial impingement.

On physical exam, the patient’s left shoulder was slightly more protracted and he had mild scapular dyskinesia. He was tender to palpation at the acromioclavicular joint, greater tuberosity, and glenohumeral joint. He actively forward elevated to 130 degrees compared to 160 degrees on the right; same with passive elevation. He internally rotated to L1 on the left and T6 on the right. He externally rotated to 70 degrees at the side bilaterally. He had full abduction which was symmetric to the other side. Internal rotation in the scapular plane was 20 degrees compared to 60 degrees on the right. External rotation in the scapular plane was 80 degrees on the left and 100 degrees on the right. Impingement tests with Neer, Hawkins, and Kim were all positive. Strength testing of all 4 rotator cuff muscles were 5/5 and symmetric. However, he had some pain with Jobe and bear hug tests. Crossbody adduction test and O’briens were positive. Instability tests were all negative and he did not have any signs of hyperlaxity per Beighton criteria. His American Shoulder and Elbow Surgeons Shoulder (ASES) score at this time was 26.6.

MRI prior to surgery reported a type VII SLAP (Snyder type II). Surgery report states that this was repaired with 2 suture anchors; 1 placed anterior to the biceps and another placed posteriorly and knots were tied. Additionally, there was a partial articular subscapularis tear that was debrided and decision to do the subpectoral biceps tenodesis was based on the subscapularis tear suggestive of biceps instability with compromise of the medial sling. The bursa was excised and a bursal sided rotator cuff tear was debrided of about 10%. MRI 1 year later shows intact superior labrum repair and biceps tenodesis. There was a progression of tendinosis, mild acromioclavicular (AC) joint arthrosis, and mild degenerative changes along the inferior glenoid with osseous spurring and mild chondral loss. X-rays showed a type III acromion with a large subacromial spur (Fig. **4a**). Based on these findings, the patient was consented for left shoulder arthroscopic subacromial decompression, distal clavicle excision, possible removal of anchors, and possible capsular release.

**Fig. (1):**
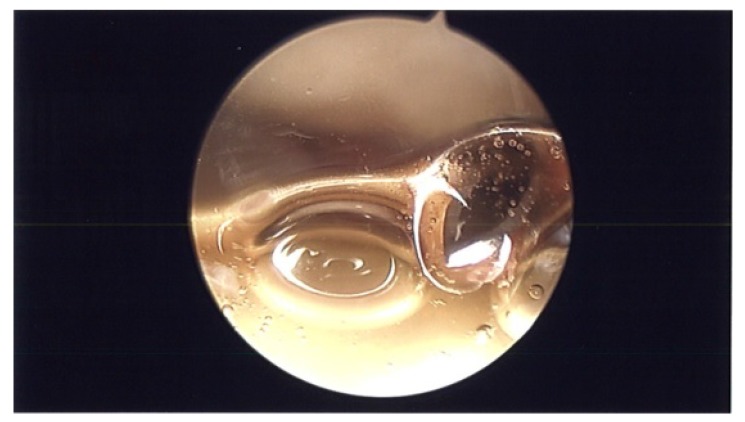
Arthroscopy picture of glenohumeral joint showing fluid that was aspirated under arthroscopic visualization.

**Fig. (3):**
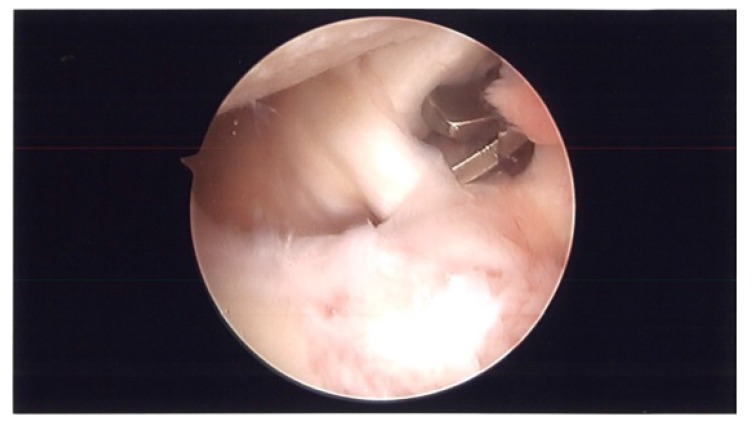
Arthroscopy picture showing resection of MGHL.

During surgery, patient was placed in lazy lateral decubitus position. Kim’s posterior portal was established. ESR and CRP were obtained pre-operatively and were negative. However, before turning on the fluid, a needle was placed in the rotator interval and intra-articular joint fluid was aspirated and sent to pathology (Fig. **1**). Prophylactic antibiotics were then started and fluid was turned on. An anterior portal was established in the rotator interval. The superior labrum had healed. There were no proud anchors and the knots were away from the articular surface. The rotator interval was thickened and scarred and the MGHL was thick and tight. The knots were removed using an open knot cutter. A superior capsular release was performed with an arthroscopic tissue liberator knife between the interval of the labrum and rotator cuff at the glenoid (Figs. **2a**-**2b**). The SGHL was released. The MGHL was resected with a meniscal punch (Fig. **3**) as well as the rotator interval and CHL. The anterior capsule had normal pliancy and was not thick and fibrotic as seen typically with adhesive capsulitis and therefore, the capsular release was not extended anteroinferiorly.

**Fig. (4a-4b):**
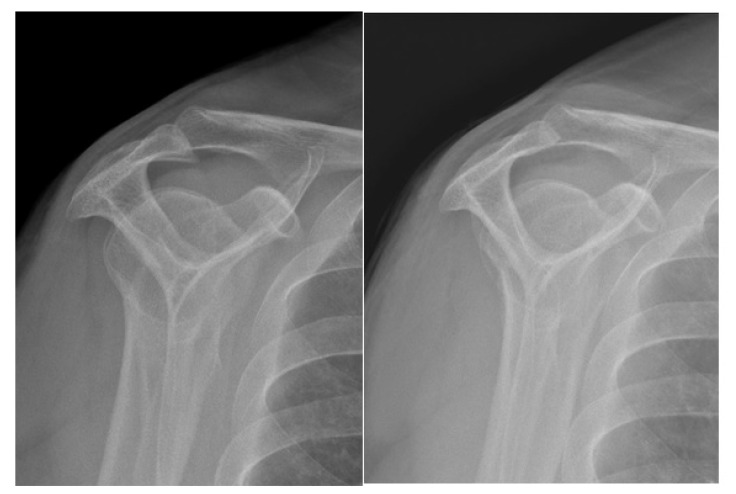
Supraspinatus outlet view of left shoulder showing type III acromion with large subacromial spur and x-ray after subacromial decompression.

In the subacromial space, there was thickened bursa and a bursectomy was performed. Adhesions were removed in the anterior, lateral, and posterior gutters. A subacromial decompression (Figs. **4a**-**b**) and distal clavicle excision were performed.

He was discharged home the same day with a sling for comfort and noted that he was able to raise his arm overhead on POD 0 which he was not able to do previously. The patient was given 3 weeks of oral penicillin until final cultures came back. He started immediate physical therapy with a range of motion exercises and periscapular strengthening and progressive cuff strengthening. Final cultures at 3 weeks were negative.

On his last follow up at 6 months post-operative, he was able to actively forward flex to 160 degrees, internally rotate to T8, externally rotate to 70 at the side, externally rotate in the scapular plane to 90 degrees, and internally rotate in the scapular plane to 60 degrees. Neer and Kim impingement tests were negative while Hawkins was mildly positive. He had symmetric strength of all four rotator cuff muscles. His final ASES score was 86.6.

## 3 DISCUSSION

This case presentation highlights several controversies that must be considered when managing a failed SLAP repair. Causes for failed SLAP repair are misdiagnosis, poor healing, recurrent injury, anchor placement, over-tensioning, proud anchors, loose taks, knots causing chondral abrasion, stiffness, and infection [4-7, 14].

Correct diagnosis of the initial SLAP tear is challenging. History and physical exam findings lack specificity [15-20]. MRI or MRA can have a high rate of false positives [21]. There is substantial inter and intraobserver variability amongst experienced shoulder arthroscopists with surgeons having difficulty distinguishing normal shoulders from type II SLAP tears [22]. Variations in biceps tendon origin on the superior labrum and variations of the anterior-superior labrum with Buford complex, sublabral foramen, sublabral sulcus can also complicate the diagnosis [23, 24]. It has been noted that there was a trend to overtreat SLAP lesions in the US and it has been postulated that some patients with a failed SLAP repair underwent repair of a normal labral variant [25, 26]. Additionally, type II SLAP tears rarely happen in isolation with one study reporting 88% incidence of coexisting pathology [27]. In our patient, workup revealed subacromial impingement and AC joint arthritis which were addressed during revision surgery.

Our patient’s main issue was stiffness. Adhesive capsulitis, over-tensioning, proud anchors and knots, arthritis, and infection were all considered. Indolent infections are one of the most common reasons for pain and stiffness in shoulder arthroplasty with Propionibacterium acne being the most commonly involved organism [28-31]. A recent study also showed that indolent infections can be a common source of pain and stiffness after shoulder arthroscopy with an incidence of 29.4% of positive cultures (23.5% for P. acnes) during revision arthroscopy for pain, stiffness, or weakness [14, 32]. Patients with P. acnes infections normally do not exhibit typical signs of infection such as fever, erythema, or a draining wound [14]. Moreover, inflammatory markers such as erythrocyte sedimentation rate and C-reactive protein are typically normal [14]. Studies have shown that these slow-growing organisms may take up to 3 weeks to culture which are longer than most protocols at hospitals [14]. Our typical algorithm is to cover patients with penicillin for 3 weeks until final cultures come back. If positive, then the treatment is an additional 3 weeks of penicillin.

During revision arthroscopy for our patient, we found no signs of hardware complications and therefore the reason for the patient’s stiffness was attributed to over-tensioning of the superior labrum. The decision to repair the superior labrum after biceps tenodesis in a nondominant arm of a 44-year-old was interesting. The superior capsule has been shown to be important in glenohumeral biomechanics limiting glenohumeral translation [33]. However, labral tears in the middle-aged patients may be the sequelae of degenerative processes similarly to age-related changes of the rotator cuff tendons [34]. Additionally, as we age, labral lesions become a less essential component to stability [35, 36]. Therefore, repairing the superior labrum in a patient without instability can over-constrain the capsulolabral complex limiting range of motion.

While nonoperative treatment with physical therapy should be tried first, there is debate on the optimal surgical treatment of type II SLAP tears [3, 8, 9, 11, 12, 27]. Initially, repair was the gold standard but some studies found suboptimal results [8, 37, 38]. Boileau found improved outcomes comparing biceps tenodesis to SLAP repair, but there was a possible age bias [8]. Subsequent studies found improved outcomes and less complications with biceps tenodesis compared to SLAP repair in patients older than 35 or 40 [9, 39, 40]. There has been a recent trend more towards biceps tenodesis over SLAP repair [8]. And with SLAP repair, there has been a debate on the optimal construct [41]. Placing an anchor anterior to the biceps origin has been shown to decrease external rotation in a cadaveric model [42]. However, Sugaya argues in throwers that putting anchors posteriorly to the biceps origin creates too tight of a structure superoposteriorly and recommends debriding the superoposterior labrum and placing one anchor anterior to the biceps to retention the MGHL to restore stability [33]. He reports a more successful return to play with elite throwers with this repair construct. But then a recent level 1 study shows no difference between biceps tenodesis, SLAP repair, and sham surgery with a community patient population [43]. The surgeon for the index procedure, in our presented patient, performed both a biceps tenodesis and superior labrum repair. Chalmers found that combined biceps tenodesis and SLAP repair do worse than either isolated biceps tenodesis or SLAP repair [13]. They performed this combined procedure for high demand patients with a SLAP tear and evidence of biceps tendonitis. They rationalized that the superior labrum may play an important role in glenohumeral stability independent of the biceps tendon. However, the authors found that combined biceps tenodesis and SLAP repair group had lower ASES scores, significantly worse forward flexion, and significantly higher pain scores. Our patient had a similar result after receiving the combined procedure, and after receiving a superior capsular release, his forward elevation normalized and his pain and ASES score significantly improved.

The CHL and MGHL have been shown to limit ER [44]. A recent biomechanical study showed that the superior capsule is important for glenohumeral stability and that sectioning the superior capsule resulted in increased glenohumeral translation in all directions particularly superior glenohumeral translation [33]. We are unaware of any biomechanical studies that examine the loss of range of motion from over constraining in the superior capsule. In our case study and the case series by Chalmers, it appears that superior labral repair can result in a decrease in forward flexion. And by releasing the superior capsule, forward elevation can be restored. Superior capsular release has previously been described for mobilizing retracted rotator cuff tears [45-48]. However, we are unaware of any reports describing superior capsular release to treat stiffness after combined SLAP repair and biceps tenodesis.

## CONCLUSION

Failed SLAP repair is often multifactorial and a thorough workup is needed. Combined biceps tenodesis and SLAP repair can cause pain, stiffness, and dysfunction which can be successfully treated with arthroscopic superior capsular release.

## Figures and Tables

**Fig. (2a):**
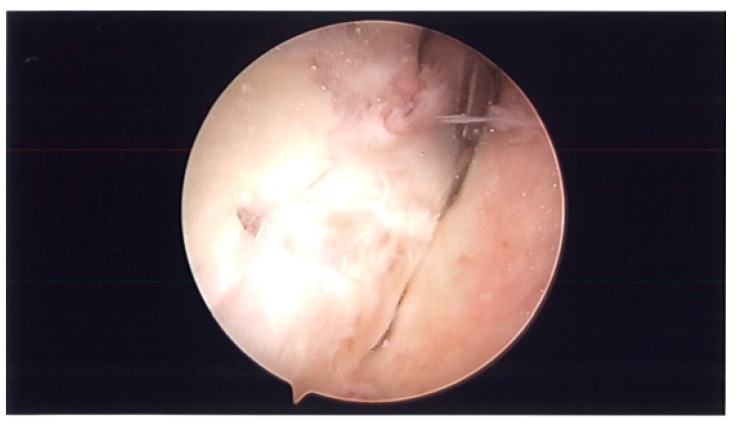
Arthroscopy picture of left shoulder showing superior capsular release with arthroscopic liberator knife.

**Fig. (2b):**
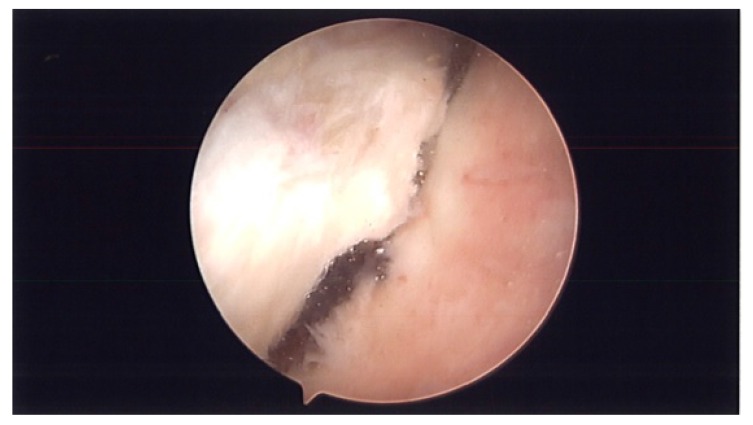
Completion of superior capsular release.

## LIST OF ABBREVIATIONS

AC: = Acromioclavicular
ASES: = American Shoulder and Elbow Surgeons Shoulder
CHL: = Coracohumeral Ligament
CRP: = C-Reactive Protein
ER: = External Rotation
ESR: = Erythrocyte Sedimentation Rate
MGHL: = Middle Glenohumeral Ligament
MRA: = Magnetic Resonance Angiogram
MRI: = Magnetic Resonance Imaging
POD: = Postoperative Day
RI: = Rotator Internal
SGHL: = Superior Glenohumeral Ligament
SLAP: = Superior Labrum Anterior and Posterior
